# Integrated chemical profiling, network pharmacology and pharmacological evaluation to explore the potential mechanism of Xinbao pill against myocardial ischaemia–reperfusion injury

**DOI:** 10.1080/13880209.2022.2025859

**Published:** 2022-02-11

**Authors:** Ying Yang, Ting Chen, Jiaming Liu, Sixuan Chen, Rongqing Cai, Liqiong Wu, Jiexiong Hu, Qiongying Lin, Xiaoxiao Qi, Zhongqiu Liu, Yuanyuan Cheng

**Affiliations:** a School of Pharmaceutical Sciences, Joint Laboratory for Translational Cancer Research of Chinese Medicine of the Ministry of Education of the People's Republic of China, International Institute for Translational Chinese Medicine, Guangzhou University of Chinese Medicine, Guangzhou, China; b Guangdong-Hong Kong-Macau Joint Lab on Chinese Medicine and Immune Disease Research, Guangzhou, China; c Research and Development Department, Guangdong Xinbao Pharm-tech Co., Ltd, Guangzhou, China; d School of Pharmacy, Guangdong Pharmaceutical University, Guangzhou, China

**Keywords:** UHPLC-QTOF-MS, chemical profile, autophagy, ER stress

## Abstract

**Context:**

Xinbao pill (XBW), a traditional Chinese herbal formula, is widely used in clinical treatment for cardiovascular diseases; however, the therapeutic effect of XBW on myocardial ischaemia–reperfusion injury (MI/RI) is unclear.

**Objective:**

This study evaluates the cardioprotective effect and molecular mechanism of XBW against MI/RI.

**Materials and methods:**

A phytochemistry-based network pharmacology analysis was used to uncover the mechanism of XBW against MI/RI. Ultra performance liquid chromatography coupled with quadrupole time-of-flight mass spectrometry method was used to identify chemicals. MI/RI-related targets of XBW were predicted using TargetNet database, OMIC database, etc. Sprague-Dawley (SD) rats under anterior descending artery ligation model were divided into Sham, MI/RI and XBW (180 mg/kg, intragastric administration). After 30 min ischaemia and 24 h reperfusion, heart tissues were collected for measurement of myocardial infarct size. After oxygen glucose deprivation for 6 h, H9c2 cells were treated with XBW (60, 240 and 720 μg/mL) and diazoxide (100 μM) for 18 h of reperfusion.

**Results:**

Thirty-seven chemicals were identified in XBW; 50 MI/RI-related targets of XBW were predicted using indicated databases. XBW significantly reduced infarct size and creatine kinase MB (CK-MB) level after MI/RI; XBW protected H9c2 cells against OGD/R injury. Gene ontology (GO) and KEGG pathway enrichment analyses by String database showed that the cardioprotective effect of XBW was associated with autophagy and apoptosis signalling pathways. Experimental investigation also verified that XBW suppressed apoptosis, autophagy and endoplasmic reticulum (ER) stress.

**Conclusions:**

XBW showed therapeutic effects against MI/RI mainly via attenuating apoptosis though suppressing excessive autophagy and ER stress.

## Introduction

Myocardial ischaemia–reperfusion injury (MI/RI) is a difficult problem after percutaneous coronary intervention (PCI) or thrombolytic therapy, which seriously affects the patients' quality of life. It is reported that about 10% patients with myocardial infarction die from lethal reperfusion injury (Yellon and Hausenloy 2007). Current mechanism studies show that MI/RI is associated with oxidative stress, inflammation, cardiomyocyte apoptosis, calcium overload or complement activation (Thind et al. 2015). Various pharmacological agents have been developed to reduce MI/RI based on them, but the effect is not ideal. It still lacks in effective and safe approaches for preventing MI/RI (Hausenloy and Yellon 2013), which force us to explore some promising therapies.

Xinbao pill (Xin-Bao-Wan, XBW) is a traditional Chinese herbal formula developed by Minghan Weng, a researcher from Guangdong Institute of Materia Medica. It consists of *Datura metel* L. (Yangjinhua), *Cornu cervi pantotrichum* (CCP, Lurong), *Aconitum carmichaelii* (Fuzi), *Panax ginseng* C.A.Mey., *Panax notoginseng* (Burk.) F.H.Chen., *Cinnamomum cassia* Presl (Rougui), moschus (Shexiang), Borneolum syntheticum (Bingpian) and Venenum Bufonis (Chansu). Xinbao pill has the effects of warming and tonifying heart and kidney; replenishing Qi and assisting Yang; promoting blood circulation to remove obstructions from meridians. In traditional Chinese medicine (TCM) clinic, Xinbao pill is used to treat chronic cardiac insufficiency caused by heart and kidney Yang deficiency; heart pulse stasis, bradycardia and sinus syndrome caused by sinus insufficiency; angina pectoris caused by ischaemic heart disease and ischaemic changes of electrocardiogram (He et al. 2020). Pharmacological studies showed that XBW and its components have a definite cardioprotection. For example, XBW suppressed cardiac hypertrophy via regulation of PI3K/Akt/GSK3β signalling pathway (He et al. 2020). XBW also attenuated chronic heart failure in a rat model (Zhao et al. 2010; Li et al. 2018). Ginsenoside Rg1, Rb1 (Zheng et al. 2017), Rg3 (Zhang et al. 2016), Rd (Zeng et al. 2015) significantly reduced myocardial infarct size and improved cardiac function in I/R injured model through suppressing oxidative stress, apoptosis and inflammation. Notoginsenoside R1 pre-treatment ameliorated myocardial injury induced by I/R via inhibiting ROCK and promoting mitochondrial ATP synthase δ-subunits (Tong et al. 2019). However, as a Chinese traditional formula, only few studies have illustrated the cardioprotection of XBW against MI/RI. Therefore, it is meaningful to clarify the underlying mechanism of XBW against MI/RI.

**Figure 1.**
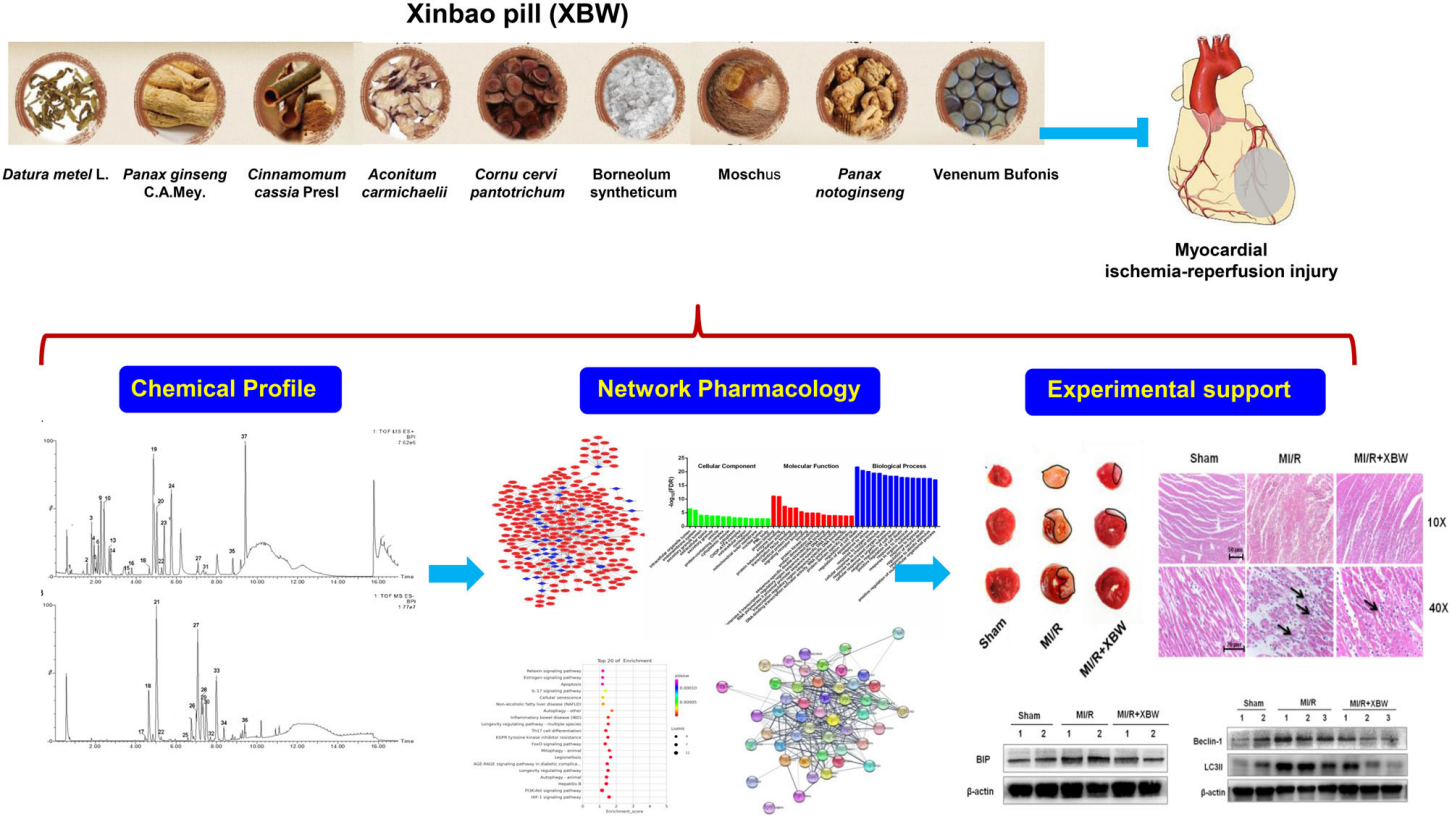
Flowchart showing the network pharmacological and experimental studies for the investigation of the cardioprotection of Xinbao pill against myocardial ischaemia–reperfusion.

This study used ultra performance liquid chromatography-quadrupole-time-of-flight tandem mass spectrometry (UPLC-Q-TOF-MS) to identify the chemical profile of XBW, and the database to find out the predicted targets of the chemicals, then built a compound-target-disease network. Finally, we systematically investigated the cardioprotective effect of XBW against MI/RI *in vitro* and *in vivo* model and verified predicted pathway by pharmacological assays (Figure 1).

## Materials and methods

### Drugs and reagents

Xinbao pills were supplied by Guangdong Xinbao Pharm-tech Co., Ltd. (Guangzhou, China). Rabbit anti-Bcl2 antibody (1:1000; Cat. no. #A00040-1) and rabbit anti-Bax antibody (1:1000; Cat. no. #BM3964) were purchased from Boster Biological Technology Co., Ltd. (Pleasanton, CA). Rabbit anti-Beclin-1 (1:1000; Cat. no. #3495), rabbit anti-LC3II antibody (1:1000; Cat. no. #2775), rabbit anti-caspase-3 antibody (1:1000; Cat. no. #9662), goat anti-rabbit or goat anti-mouse IgG-horseradish peroxidase (HRP)-conjugated secondary antibodies (1:3000, Cat. no. #7074 or #7076) were from Cell Signaling Technology (Boston, MA). Rabbit anti-β-tubulin antibody (1:1000, Cat. no. #bs0210R) was bought from Beijing Biosynthesis Biotechnology Co., Ltd. (Beijing, China). Mouse anti-GAPDH antibody (1:1000; Cat. no. #GB12002) and mouse anti-β-actin antibody (1:1000, Cat. no. #GB12001) were purchased from Servicebio (Wuhan, China). Diazoxide (Cat. no. #D9035), 3-(4,5-dimethylthiazol-2-yl)-2,5-diphenyltetrazolium bromide (MTT, Cat. no. #M5655) and triphenyltetrazolium chloride (TTC, Cat. no. #T8877) were obtained from Sigma-Aldrich (St. Louis, MO).

### Ultra performance liquid chromatography-quadrupole-time-of-flight tandem mass spectrometry analysis

Xinbao pills were first broken into powder. A total of 1.0 g of powder was weighted and extracted with 30 mL of methanol solution by ultrasonic extraction for 30 min twice. Extracted solutions were combined and concentrated. After that, it was prepared into 10 mg/mL stock solution and filtered by 0.45 μm micropore film for analysis. UPLC-Q-TOF-MS analysis was performed with Waters Acquity UPLC I-class and a Xevo G2-S Q Tof time-of-flight mass spectrometer (Waters Corporation, Milford, MA). Waters BEH C18 column (2.1 × 100 mm, 1.7 μm) was used for separation. The mobile phase consisted of water containing 0.1% formic acid (A) and acetonitrile (B). The gradient was used as follows: 0.00–0.5 min, 5% B; 0.5–1.0 min, 5–15% B; 1.0–4.0 min, 15–25% B; 4.0–6.0 min, 25–35% B; 6.0–8.0 min, 35–45% B; 8.0–9.0 min, 45–75% B; 9.0–11.0 min, 75–90% B; 11.0–13.0 min, 90–95% B; 13.0–15.0 min, 95% B; 15.0–15.1 min, 95–5% B; 15.1–17.0 min, 5% B. The column oven was set at 45 °C, the flow rate was 0.4 mL/min and the injection volume was 0.5 μL. Optimal MS parameters were set as follows: the ion source temperature (120 °C); the capillary voltage (2.0 kV); the cone voltage (40 V); the desolvation gas temperature (400 °C); the desolvation gas flow (800 L/h). Masslynx 4.1 software was used to analyse the data and the Waters UNIFI Scientific Information System was used to process the structure of the chemical compositions.

### Cells and treatment

Rat cardiomyocyte H9c2 cells were purchased from the American Type Culture Collection (ATCC, Manassas, VA). H9c2 cells were cultured in DMEM including 10% foetal bovine serum (FBS) and 1% penicillin/streptomycin (PS) at 37 °C, 5% CO_2_. For treatment, H9c2 cells were treated with indicated drugs (XBW (0, 10, 60, 240 and 720 μg/mL), diazoxide (100 μM)) and then subjected to OGD condition (after medium was washed by phosphate buffer solution (PBS) and replaced with DMEM without glucose and FBS, cells were put into a chamber saturated with 95% N_2_ and 5% CO_2_) for 6 h and reperfusion for another 18 h. For drug preparation, powdered XBW was extracted with ethanol by ultrasonic extraction for 30 min twice. After concentration, extracts were dissolved in DMEM containing 0.5% ethanol and filtered by 0.22 μm to prepare different doses given drugs.

### Cell viability

The cell viability was measured by the MTT assay. In brief, after drug treatment, 0.5 mg/mL MTT solution was added to 96-well plate and incubated at 37 °C for 4 h. When removing the supernatant, 150 μL of dimethyl sulphoxide (DMSO) was used to dissolve the formazan products. The absorption was determined by a microplate reader at 490 nm (Thermo Fisher Scientific, Waltham, CA).

### Western blot analysis

Briefly, H9c2 cells and cardiac tissue were extracted by 1× RIPA buffer (Solarbio Life Sciences, Beijing, China) containing protease and phosphatase inhibitors. The proteins were loaded to 10% SDS-polyacrylamide gels for electrophoresis and transferred to a polyvinylidene difluoride (PVDF) membrane. After blotting in 5% non-fat milk for 1.0 h, the membranes were incubated with specific primary antibodies (Bcl2, Caspase-3, Bax, Baclin-1, LC3II, BIP, GAPDH, β-tubulin, β-actin) overnight and goat anti-rabbit or goat anti-mouse IgG-HRP-conjugated secondary antibodies for 1 h at room temperature (RT). After washing with 1× TBST, the bands were detected with enhanced chemiluminescence (ECL) detection reagents from Absin Bioscience Inc. (Shanghai, China).

### Animal experiments

The experimental procedures and protocols were approved by the Committee on Ethical USE of Animals of Guangzhou University of Chinese Medicine (no. IITCM-20180306). Sprague-Dawley (SD) male rats (weighting 250–280 g) were obtained from Animal Laboratory Center of Southern Medical University and divided into three groups: Sham group (*n* = 6), MI/R model group (MI/R + saline, *n* = 6) and XBW group (MI/R + XBW, *n* = 6). In brief, SD rats were anaesthetized by intraperitoneal injection of 40 mg/kg 2% pentobarbital sodium. After anaesthesia, rats were fixed and plugged into a ventilator. After opening the chest between the 3rd and 4th rib, the left anterior descending (LAD) artery was ligated with a 6-0 silk suture and PE10 tube for 30 min, and then reperfusion for 24 h. Sham group only received left thoracotomy without ligation. The MI/R + XBW group received 180 mg/kg XBW by intragastric administration at 1 h before MI/R. Powdered XBW was dissolved in 0.9% saline, and prepared the given solution according to the weight of rats. After 24 h of reperfusion, heart tissues were cut into 2 mm sections, and incubated in 1% TTC solution at 37 °C for 15 min, then fixed in 4% paraformaldehyde (PFA) overnight. The images of these sections were captured, and the infarct size was analysed and calculated by image J (NIH Image J system, Bethesda, MD).

### H&E staining

Hearts were fixed in the 4% PFA solution, dehydrated by gradient alcohol, and embedded with paraffin. Paraffin slices (5 μm) were rehydrated by gradient alcohol, and stained with haematoxylin and eosin (H&E). Then, the slices were washed with water, dehydrated by 80%, 90% and 100% ethanol, and sealed with neutral gum. Finally, the slices were observed and photographed under light microscope.

### Predicting targets of compounds in XBW

The mol2 format files of UPLC-Q-TOF-MS-identified chemicals were downloaded from Pubchem database and uploaded to TargetNet webserver (http://targetnet.scbdd.com). In practice, protein targets with prediction score of >0.90 were selected. In addition, the targets of chemicals reported in literature were also collected.

### Collecting MI/RI-associated targets

The targets related to MI/RI were selected from OMIM database (https://omim.org/) and the literature using ‘myocardial ischemia–reperfusion injury’ as keywords.

### Gene ontology (GO) and pathway enrichment of potential targets

The GO, biological process (BP), molecular function (MF) and cellular component (CC), Kyoto Encyclopedia of Genes and Genomes (KEGG) signalling pathway were analysed by using the String Database (https://string-db.org/cgi/input.pl). Only the false discovery rate (FDR) ≤0.05 was selected.

### Statistics analysis

The results were expressed as means ± SD or means ± SEM from no less than three independent experiments. Statistical analysis was performed by one-way ANOVA with GraphPad Prism software (La Jolla, CA). A *p* value <0.05 was considered as statistical significant.

## Results

### Chemical profile of XBW by UPLC-QTOF-MS

**Figure 2.**
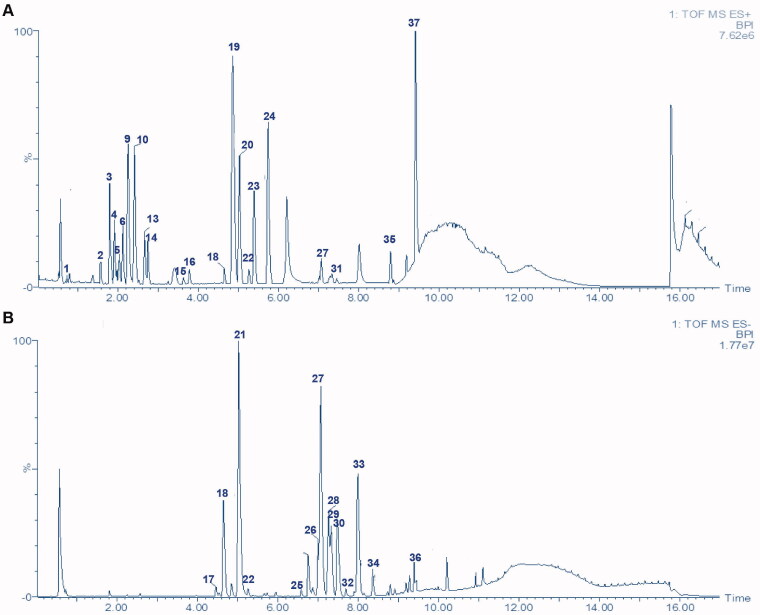
Identification of chemical constituents from XBW extracts (A) positive ionization mode; (B) negative ionization mode.

A specific UPLC-QTOF-MS method was used to identify the chemicals in XBW. As shown in Figure 2 and Table 1, a total of 37 compounds were identified by comparing their retention time with that of reference compounds or comparing their retention behaviours, empirical molecular formula and proposed fragmentations with that in literature (Takayama et al. 1990; Boermans et al. 2006; Wu et al. 2012; Wang et al. 2014; Chen DX et al. 2015; Chen YJ et al. 2015; Xu et al. 2017; Chen et al. 2018; Cirlini et al. 2018; Du et al. 2018; Gong et al. 2019; Shen et al. 2019; Zhang et al. 2019; Wei et al. 2020; Yao et al. 2021), including ginsenoside Rg1-3, Rb1-3, Re, Ro, gypenoside XVII, aconine, mesaconine, isotalatizidine, scopolamine, arenobufagin, etc. The chemical components were derived from Fuzi, *Panax ginseng*, *Panax notoginseng*, *Datura metel* L., CCP and Chansu in XBW.

**Table 1.** Characterization of the chemical constituents in XBW by UHPLC-Q-TOF-MS.

No.	Chemical name	Pubchem CID	Cas no.	Formula	2D structure	Molecular weight (Da)	ESI+ (*m/z*)	ESI– (*m/z*)	Fragmentations (*m/z*)	RT (min)	Chinese Medicine	Ref.
1	Adenosine	60961	58-61-7	C_10_H_13_N_5_O_4_	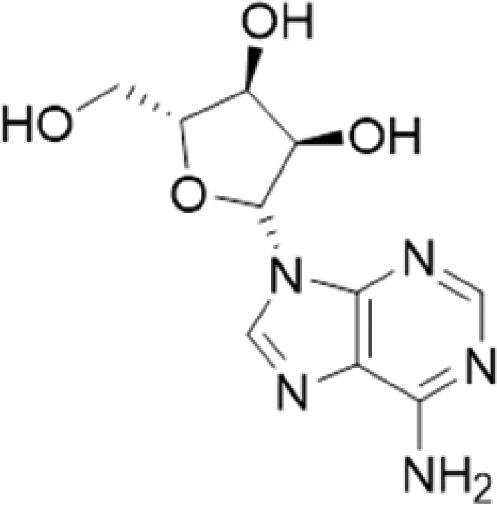	267.0967	[M + H]^+^: 268.1047	[M–H]^–^: 266.1047	136.0609, [M + H-C_5_H_8_O_4_]^+^	0.80	CCP	Shen et al. (2019)
2	Karakolidine	101306844	41655-13-4	C_22_H_35_NO_5_	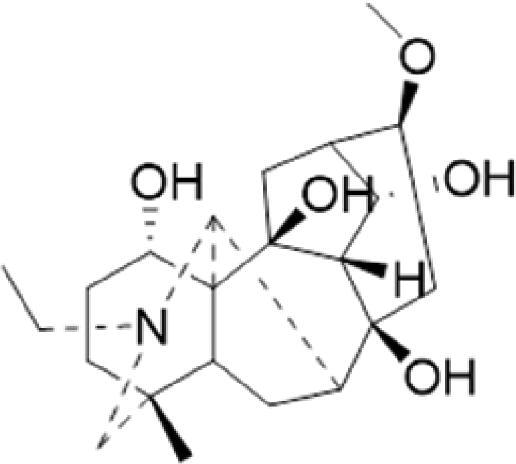	393.2515	[M + H]^+^: 394.2594	–	376.2474 [M + H-H_2_O]^+^; 317.1795 [M + H-H_2_O-C_3_H_9_N]^+^	1.58	Fuzi	Zhang et al. (2019)
3	Mesaconine	101671037	6792-09-2	C_24_H_39_NO_9_	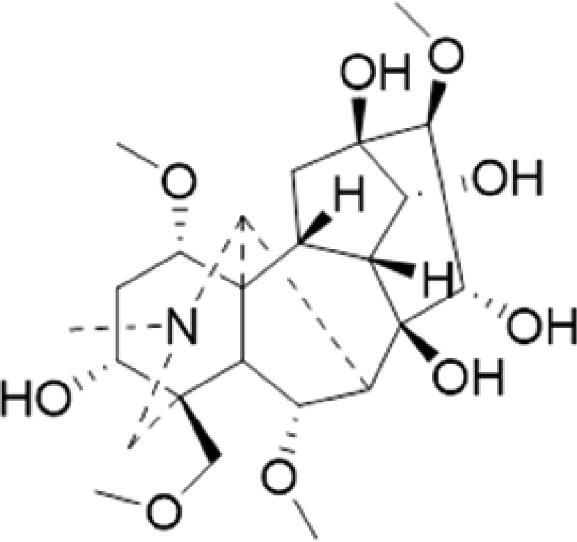	485.2625	[M + H]^+^: 486.2716		468.2616 [M + H-H_2_O]^+^; 454.2431 [M + H-H_2_O-CH_2_]^+^; 424.2740 [M + H-H_2_O-CH_2_-CH_2_O]^+^	1.80	Fuzi	Zhang et al. (2019)
4	Isotalatizidine	11452543	7633-68-3	C_23_H_37_NO_5_	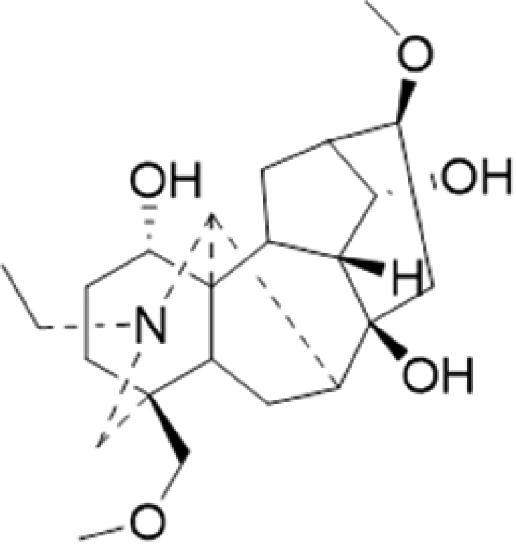	408.2730	[M + H]^+^: 408.2730		390.2634 [M + H-H_2_O]^+^; 378.2625 [M + H-CH_2_O]^+^; 360.2511 [M + H-H_2_O-CH_2_O]^+^	1.91	Fuzi	Zhang et al. (2019)
5	Aconine	417761	509-20-6	C_25_H_41_NO_9_	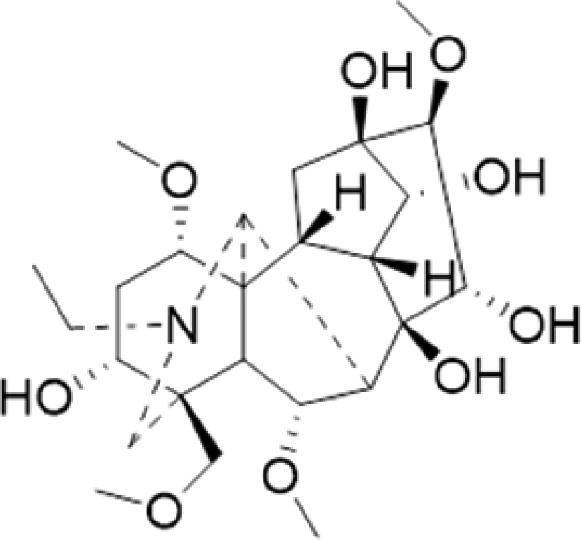	499.2781	[M + H]^+^: 500.2869		468.2616 [M + H-H_2_O-CH_2_]^+^; 454.2780 [M + H-H_2_O-C_2_H_4_]^+^; 438.2846 [M + H-H_2_O-CH_2_-CH_2_O]^+^; 408.2730 [M + H-H_2_O-CH_2_-CH_2_O-C_2_HO]^+^; 378.2625 [M + H-H_2_O-CH_2_-CH_2_O-CH_2_O]^+^	1.97	Fuzi	Zhang et al. (2019)
6	Songorine	139291804	509-24-0	C_22_H_31_NO_3_	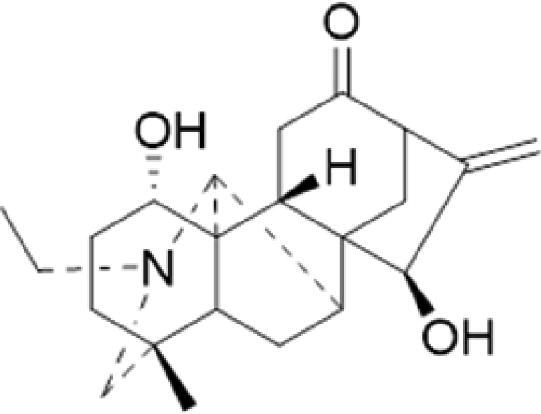	357.2304	[M + H]^+^: 358.2383		340.2252 [M + H-H_2_O]^+^; 330.2276 [M + H-C_2_H_4_]^+^	2.04	Fuzi	Zhang et al. (2019)
7	Scopolamine	3000322	51-34-3	C_17_H_21_NO_4_	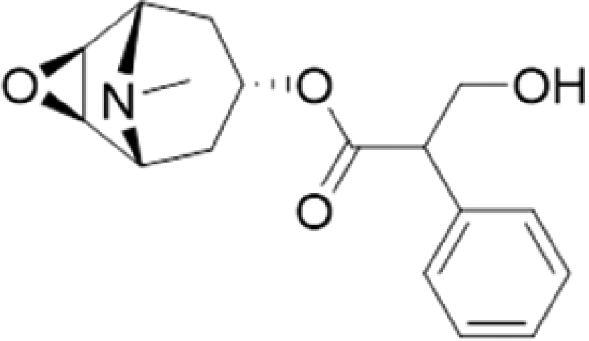	303.1471	[M + H]^+^: 304.1531		156.1013 [M + H-C_9_H_8_O_2_]^+^; 138.0900 [M + H-C_9_H_8_O_2_-H_2_O]^+^	2.13	*Datura metel* L.	Cirlini et al. (2018)
8	Hypaconine	101671038	63238-68-6	C_24_H_39_NO_8_	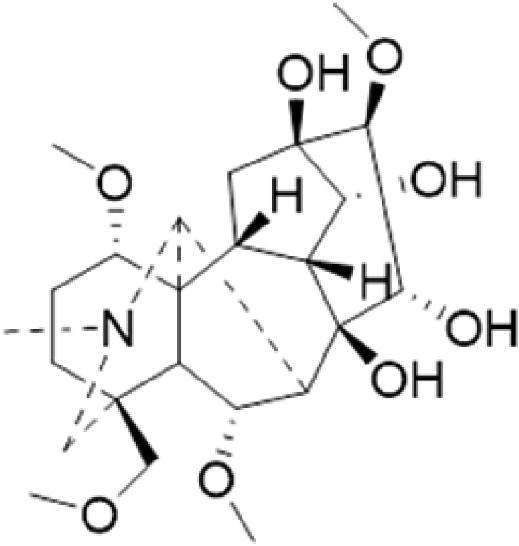	469.2676	[M + H]^+^: 470.2740		438.2520 [M + H-CH_3_OH]^+^	2.23	Fuzi	Zhang et al. (2019)
9	Fuziline	131675180	80665-72-1	C_24_H_39_NO_7_	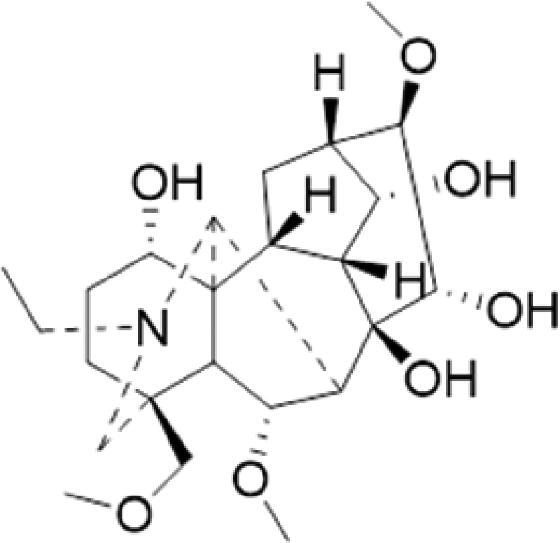	453.2726	[M + H]^+^: 454.2824	[M + HCOO]^–^: 498.2682	436.2692 [M + H-H_2_O]^+^	2.26	Fuzi	Zhang et al. (2019)
10	Neoline	12313185	466-26-2	C_24_H_39_NO_6_	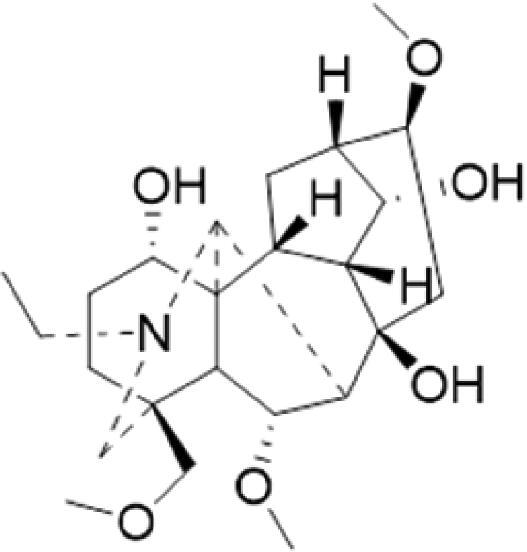	437.2777	[M + H]^+^: 438.2846		420.2758 [M + H-H_2_O]^+^; 388.2489 [M + H-H_2_O-CH_3_OH]^+^	2.41	Fuzi	Zhang et al. (2019)
11	10-Hydroxyneoline	138114026	132362-42-6	C_24_H_39_NO_7_	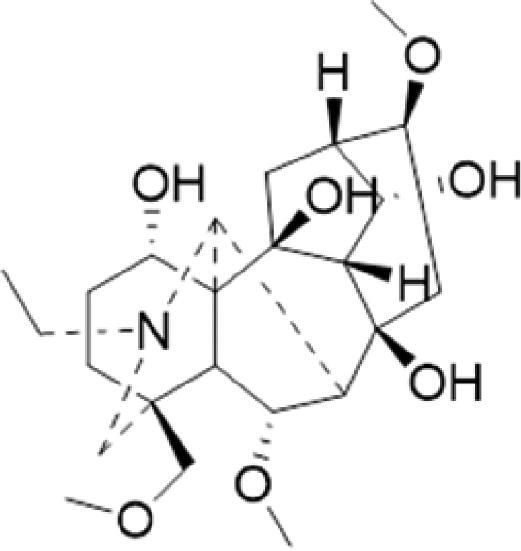	453.2726	[M + H]^+^: 454.2824		438.2468 [M + H-H_2_O]^+^; 406.2579 [M + H-H_2_O-CH_3_OH]^+^	2.52	Fuzi	Takayama et al. (1990)
12	3-Deoxyaconine	132580133	5877-69-0	C_25_H_41_NO_8_	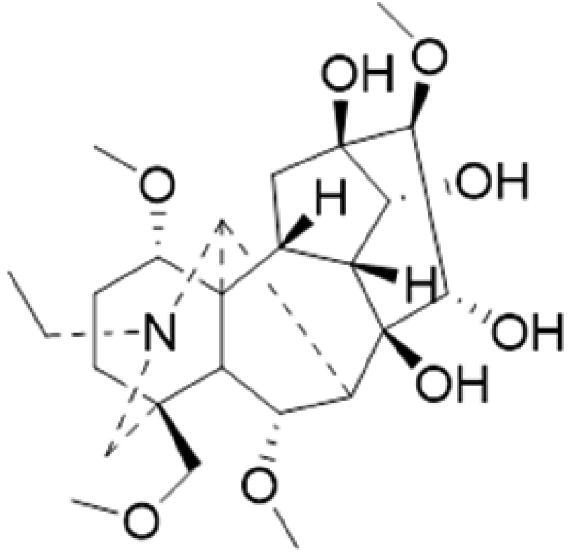	483.2832	[M + H]^+^: 484.2882		452.2610 [M + H-C_2_H_4_]^+^	2.59	Fuzi	Wang et al. (2014)
13	Atropine	174174	51-55-8	C_17_H_23_NO_3_	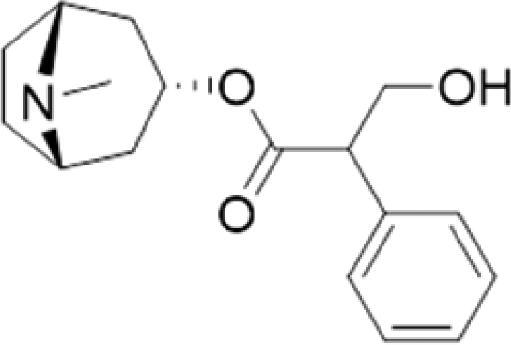	289.1678	[M + H]^+^: 290.1751		260.1735 [M + H-CH_2_O]^+^	2.66	*Datura metel* L.	Boermans et al. (2006)
14	Talatisamine	159891	20501-56-8	C_24_H_39_NO_5_	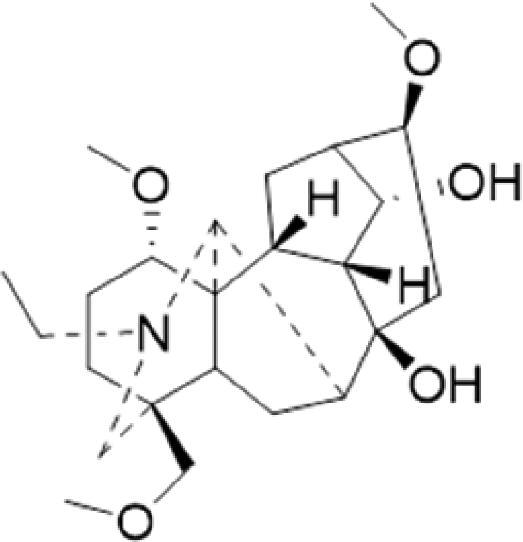	421.2828	[M + H]^+^: 422.2917		390.2634 [M + H-CH_3_OH]^+^; 372.2533 [M + H-CH_3_OH-H_2_O]^+^	2.74	Fuzi	Zhang et al. (2019)
15	14-Acetyltalatizamine	156166	71239-55-9	C_26_H_41_NO_6_	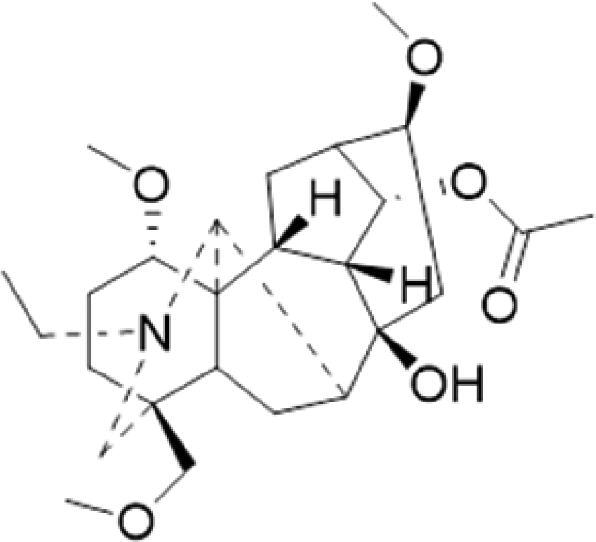	463.2934	[M + H]^+^: 464.3026		432.2732 [M + H-CH_3_OH]^+^	3.64	Fuzi	Zhang et al. (2019)
16	14-Benzoyl-10 -hydroxymesaconine	70692815		C_31_H_43_NO_11_	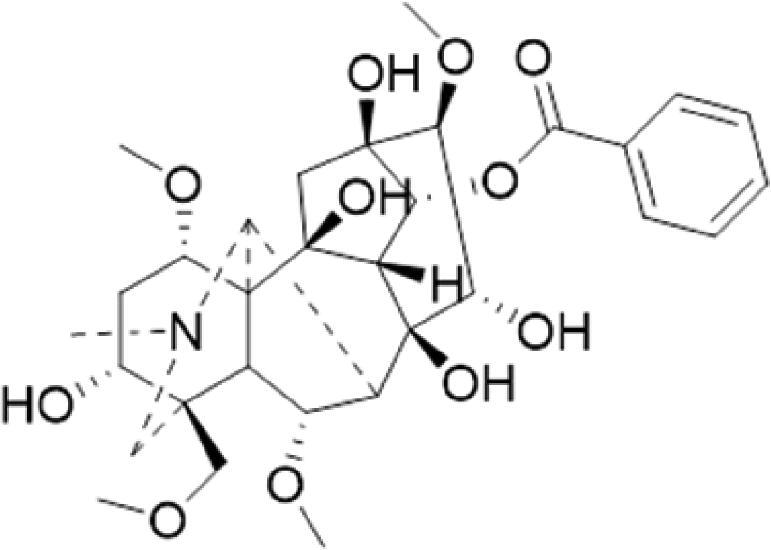	605.2836	[M + H]^+^: 606.2900		588.2823 [M + H-H_2_O]^+^; 556.2526 [M + H-H_2_O-CH_3_OH]^+^	3.79	Fuzi	Wu et al. (2012)
17	Ginsenoside M6A	90478300	93376-72-8	C_48_H_82_O_19_	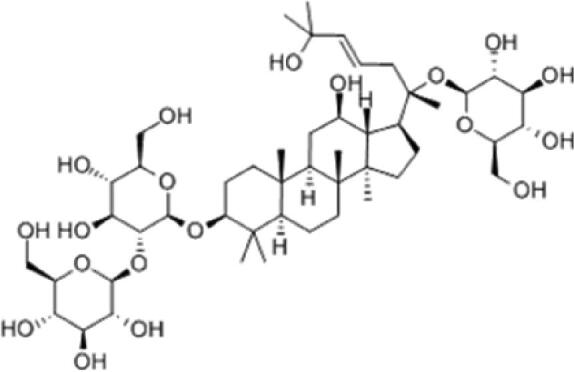	952.5450		[M–H]^–^: 951.5444; [M + HCOO]^–^: 1007.5488	799.4877 [M–H-glu]^–^; 637.4298 [M–H-glu-glu]^–^; 475.3797 [M–H-glu-glu-glu]^–^	4.47	*Panax ginseng* C.A.Mey.	Li et al. (2019)
18	Notoginsenoside R1	441934	80418-24-2	C_47_H_80_O_18_	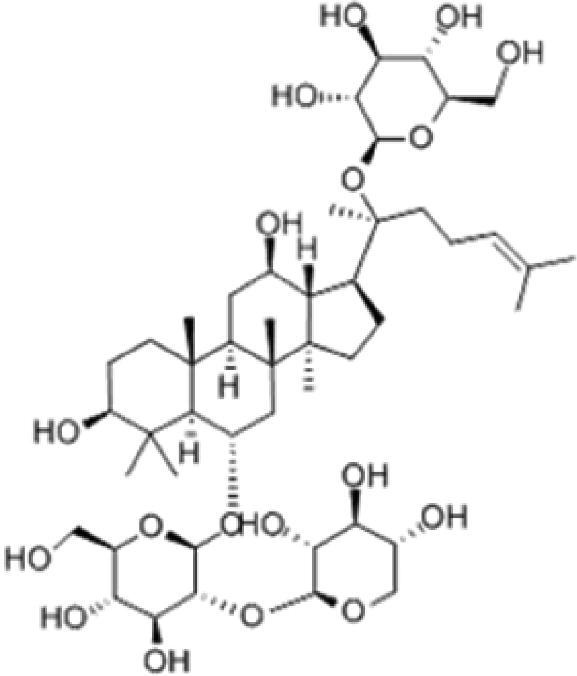	932.5345	[M + Na]^+^: 955.5221	[M–H]^–^: 931.5304; [M + HCOO]^–^: 977.5429	799.4877 [M–H-xyl]^–^; 637.4356 [M–H-xyl-glu]^–^; 475.3797 [M–H-xyl-glu-glu]	4.65	*Panax notoginseng*	Chen et al. (2018)
19	Benzoylmesaconine	24832659	63238-67-5	C_31_H_43_NO_10_	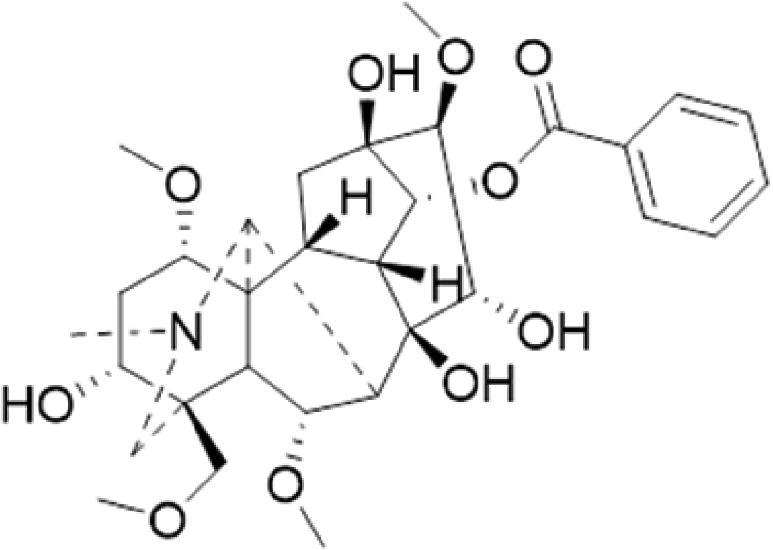	589.2887	[M + H]^+^: 590.2991		558.2717 [M + H-CH_3_OH]^+^; 540.2599 [M + H-CH_3_OH-H_2_O]^+^; 526.2453 [M + H-CH_3_OH-CH_3_OH]^+^; 508.2315 [M + H-CH_3_OH-CH_3_OH-H_2_O]^+^	4.86	Fuzi	Zhang et al. (2019)
20	Ginsenoside Rg1	441923	22427-39-0	C_42_H_72_O_14_	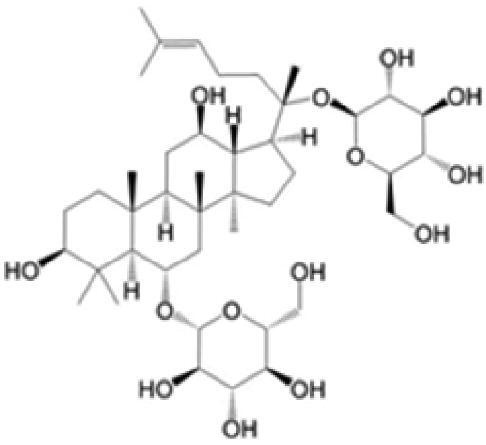	800.4922	[M + Na]^+^: 823.4822	[M–H]^–^: 799.4847; [M + HCOO]^–^: 845.5045	637.4375 [M–H-glu]^–^; 475.3797 [M–H-glu-glu]^–^	5.03a	*Panax ginseng* C.A.Mey.	Chen et al. (2018)
21	Ginsenoside Re	441921	52286-59-6	C_48_H_82_O_18_	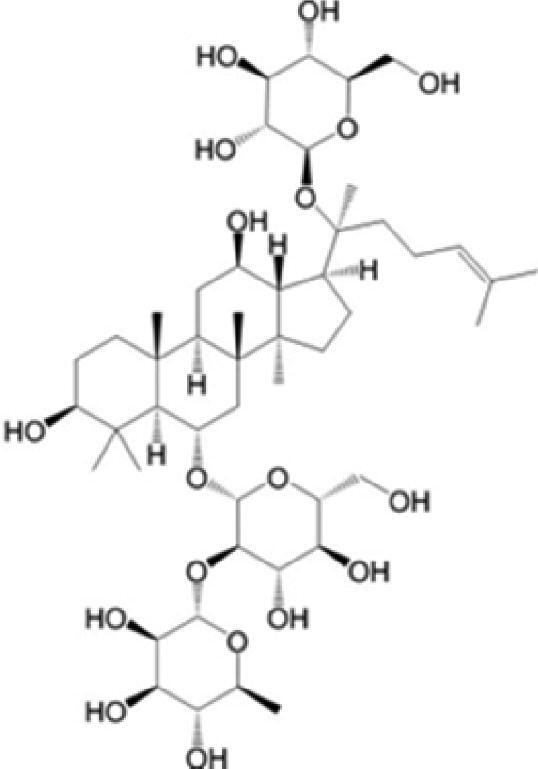	946.5501	[M + Na]^+^: 969.5352	[M–H]^–^: 945.5521; [M + HCOO]^–^: 991.5571	799.4847 [M–H-Rha]^–^; 637.4375 [M–H-Rha-glu]^–^	5.03b	*Panax ginseng* C.A.Mey.	Chen DX et al. (2015)
22	Arenobufagin	12305198	464-74-4	C_24_H_32_O_6_	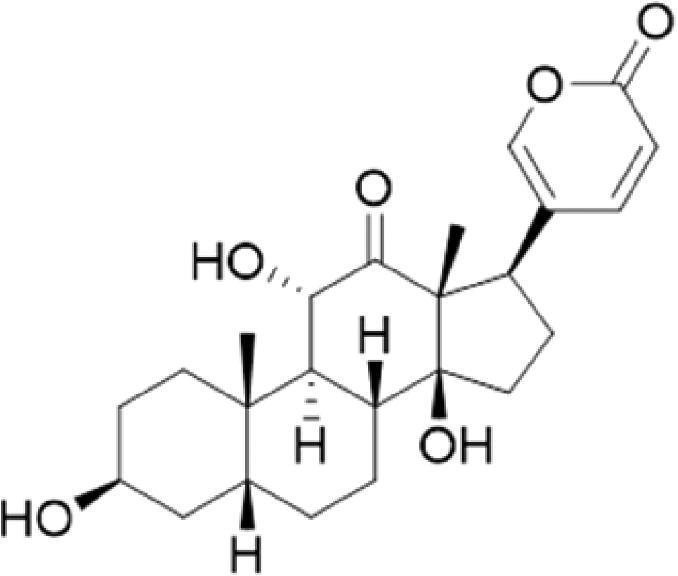	416.2199	[M + H]^+^: 417.2274		399.2154 [M + H-H_2_O]^+^; 371.2236 [M + H-H_2_O-H_2_O]^+^	5.26	Chansu	Wei et al. (2020)
23	Benzoylaconine	20055771	466-24-0	C_32_H_45_NO_10_	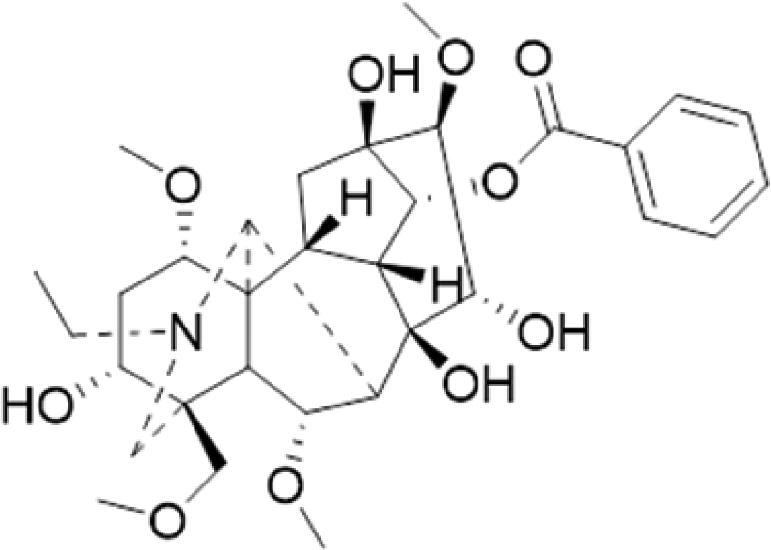	603.3043	[M + H]^+^: 604.3115		586.3038 [M + H-H_2_O]^+^; 572.2827 [M + H-CH_3_OH]^+^; 554.2757 [M + H-CH_3_OH-H_2_O]^+^; 522.2489 [M + H-CH_3_OH-H_2_O-CH_3_OH]^+^	5.39	Fuzi	Zhang et al. (2019)
24	Benzoylhypaconine	78358526	63238-66-4	C_31_H_43_NO_9_	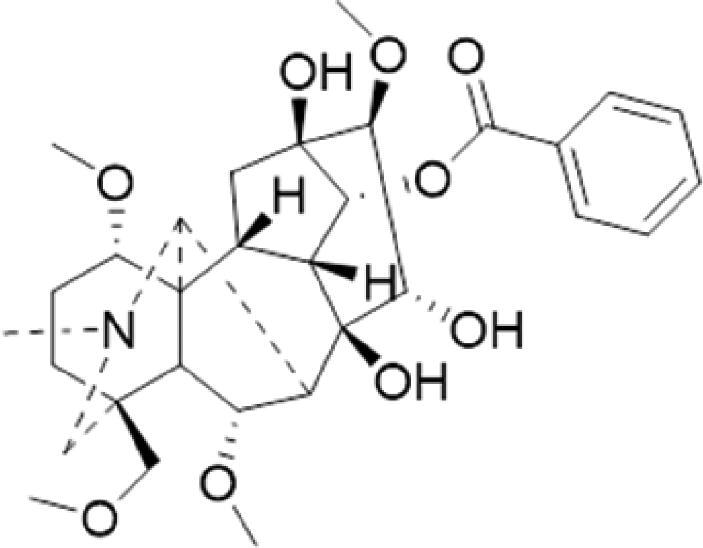	573.2938	[M + H]^+^: 574.3012	[M + HCOO]^–^: 618.2905	542.2737 [M + H-CH_3_OH]^+^; 510.2493 [M + H-CH_3_OH-CH_3_OH]^+^	5.74	Fuzi	Zhang et al. (2019)
25	Ginsenoside Ra3	73157064	90985-77-6	C_59_H_100_O_27_	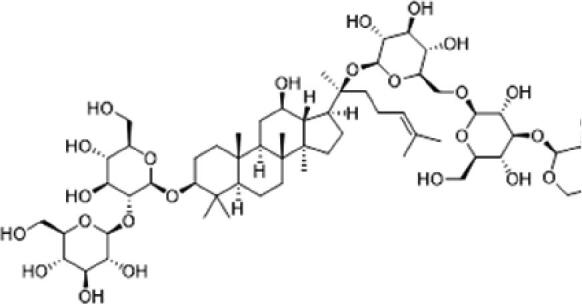	1240.6451		[M–2H]/2–: 619.3145	1107.6091 [M–H-xyl]^–^; 945.5458 [M–H-xyl-glu]^–^; 783.4946 [M–H-xyl-glu-glu]^–^	6.59	*Panax ginseng* C.A.Mey.	Chen YJ et al. (2015)
26	Ginsenoside F3	46887678	62025-50-7	C_41_H_70_O_13_	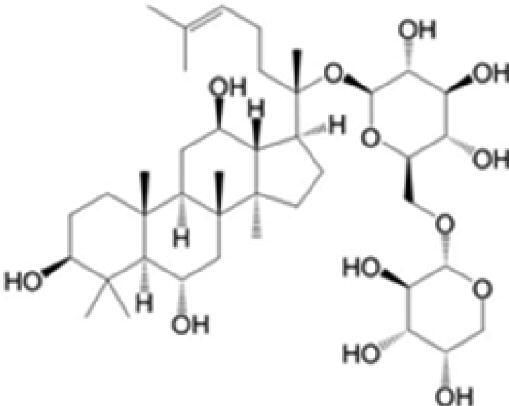	770.4816		[M–H]^–^: 769.4797; [M + HCOO]^–^: 815.4846	637.4350 [M–H-glu]^–^; 475.3797 [M–H-glu-glu]^–^	7.00	*Panax ginseng* C.A.Mey.	Du et al. (2018)
27	Ginsenoside Rb1	9898279	41753-43-9	C_54_H_92_O_23_	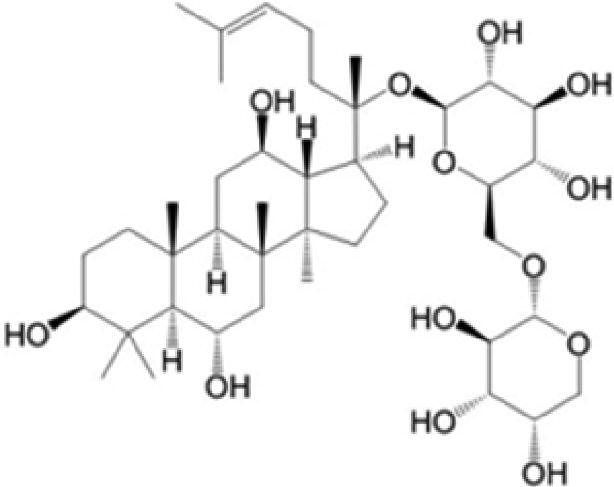	1108.6029	[M + Na]^+^: 1139.5986	[M–H]^–^: 1107.6023; [M + HCOO]^–^: 1153.6107	945.5458 [M–H-glu]^–^; 783.4946 [M–H-glu-glu]^–^; 621.4415 [M–H-glu-glu-glu]^–^	7.07	*Panax ginseng* C.A.Mey.	Chen DX et al. (2015)
28	Ginsenoside Rb2	432450	11021-13-9	C_53_H_90_O_22_	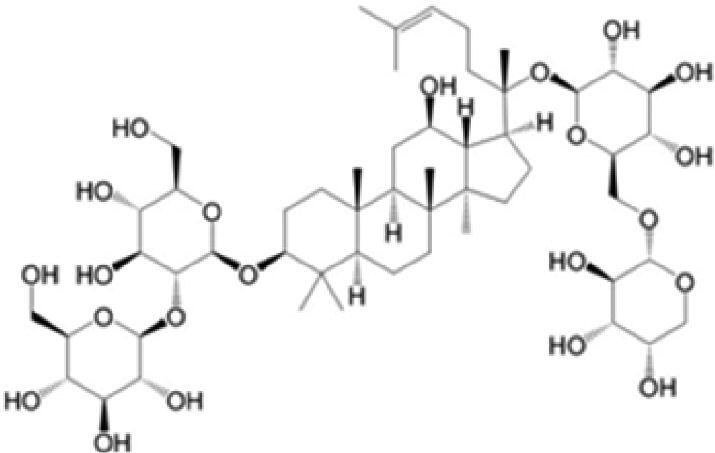	1078.5924		[M–H]^–^: 1077.5958; [M + HCOO]^–^: 1123.6008	945.5458 [M–H-Ara]^–^; 783.4946 [M–H-Ara-glu]^–^	7.27	*Panax ginseng* C.A.Mey.	Chen DX et al. (2015)
29	Ginsenoside Ro	11815492	34367-04-9	C_48_H_76_O_19_	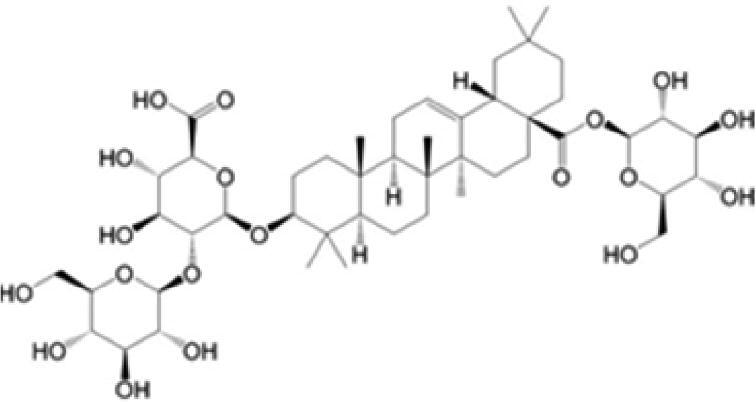	956.4981		[M–H]^–^: 955.4960	793.4425 [M–H-glu]^–^	7.34	*Panax ginseng* C.A.Mey.	Du et al. (2018)
30	Ginsenoside Rb3	12912363	68406-26-8	C_53_H_90_O_22_	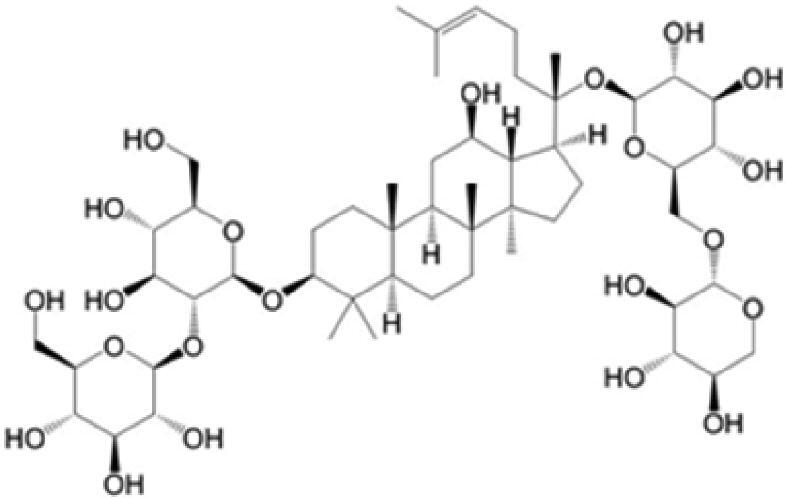	1078.5924		[M–H]^–^: 1077.5958; [M + HCOO]^–^: 1123.6008	945.5458 [M–H-xyl]^–^; 783.4946 [M–H-xyl-glu]^–^	7.48a	*Panax ginseng* C.A.Mey.	Chen YJ et al. (2015)
31	Ginsenoside Rc	12855889	11021-14-0	C_53_H_90_O_22_	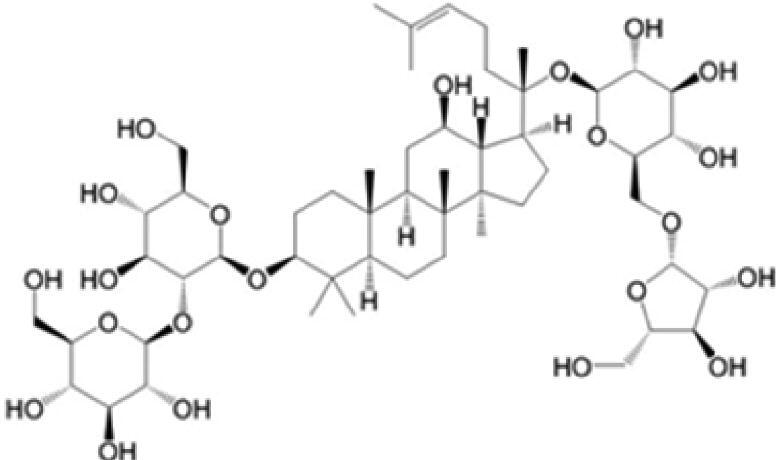	1078.5924		[M–H]^–^: 1077.5958; [M + HCOO]^–^: 1123.6008	945.5458 [M–H-Xylofuranose]^–^; 783.4946 [M–H-Xylofuranose-glu]^–^	7.48b	*Panax ginseng* C.A.Mey.	Chen et al. (2018)
32	Ginsenoside b1	71587485	132929-86-3	C_56_H_94_O_24_	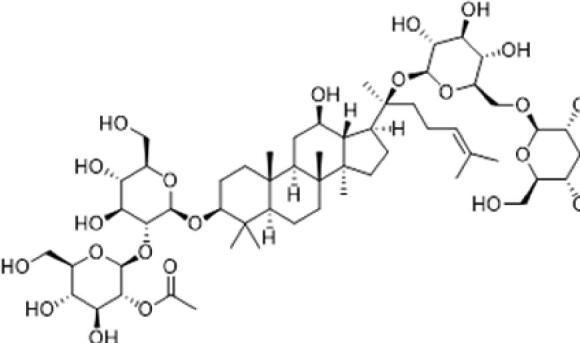	1150.6135		[M–H]^–^: 1149.6147; [M + HCOO]^–^: 1195.6218	1107.6023 [M–H-Ac]^–^; 1089.5950 [M–H-Ac-H_2_O]^–^; 1077.5891 [M–H-Ac-CH_2_O]^–^; 945.5458 [M–H-Ac-Glu]^–^	7.70	*Panax ginseng* C.A.Mey.	
33	Ginsenoside Rd	24721561	52705-93-8	C_48_H_82_O_18_	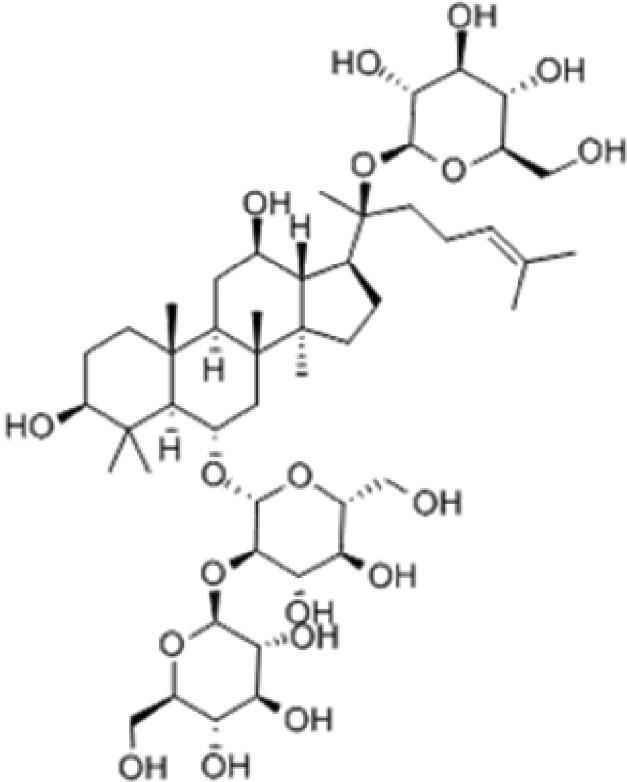	946.5501		[M–H]^–^: 945.5458; [M + HCOO]^–^: 991.5571	783.4946 [M–H-glu]^–^; 621.4415 [M–H-glu-glu]^–^	8.00	*Panax ginseng* C.A.Mey.	Chen et al. (2018)
34	Gypenoside XVII	44584555	80321-69-3	C_48_H_82_O_18_	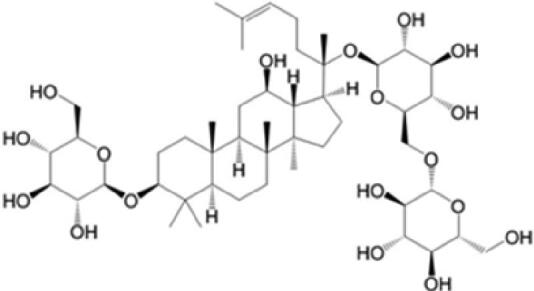	946.5501		[M–H]^–^: 945.5458; [M + HCOO]^–^: 945.5458	783.4946 [M–H-glu]^–^; 621.4415 [M–H-glu-glu]^–^	8.36	*Panax ginseng* C.A.Mey.	Xu et al. (2017)
35	Acetyl ginsenoside Rd	73818238	102805-32-3	C_50_H_84_O_19_	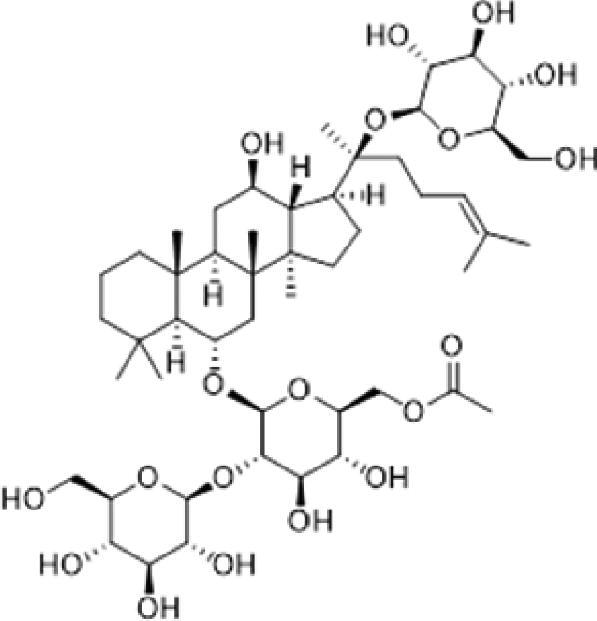	988.5607		[M–H]^–^: 987.5566; [M + HCOO]^–^: 1033.5660	945.5458 [M–H-Ac]^–^; 927.5345 [M–H-Ac-H_2_O]^–^; 783.4946 [M–H-Ac-glu]^–^	8.72	*Panax ginseng* C.A.Mey.	Yao et al. (2021)
36	Ginsenoside Rg2	21599924	52286-74-5	C_42_H_72_O_13_	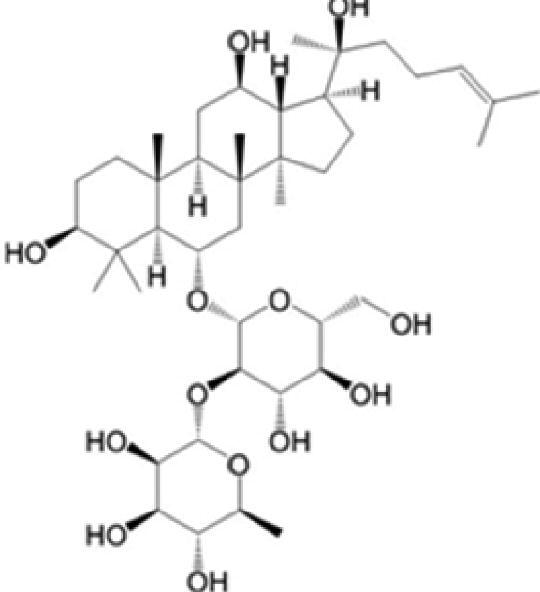	784.4973		[M–H]^–^: 783.4946; [M + HCOO]^–^: 829.4975	621.4363 [M–H-glu]^–^; 459.3836 [M–H-glu-glu]^–^	9.39	*Panax ginseng* C.A.Mey.	Chen et al. (2018)
37	Ginsenoside Rg3	9918693	14197-60-5	C_42_H_72_O_13_	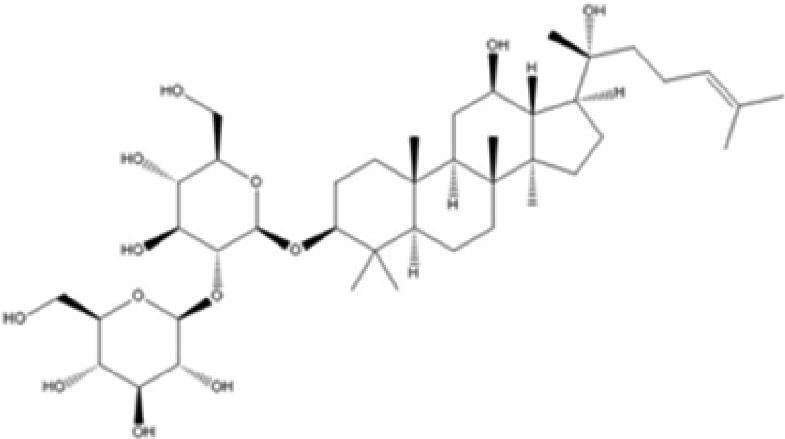	784.4973		[M–H]^–^: 783.4946; [M + HCOO]^–^: 829.4975	621.4363 [M–H-glu]^–^; 459.3836 [M–H-glu-glu]^–^	9.43	*Panax ginseng* C.A.Mey.	Chen et al. (2018)

### Effect of XBW against myocardial ischaemia–reperfusion injury in rat model *in vivo* and oxygen glucose deprivation-reperfusion (OGD/R) cell model *in vitro*

**Figure 3.**
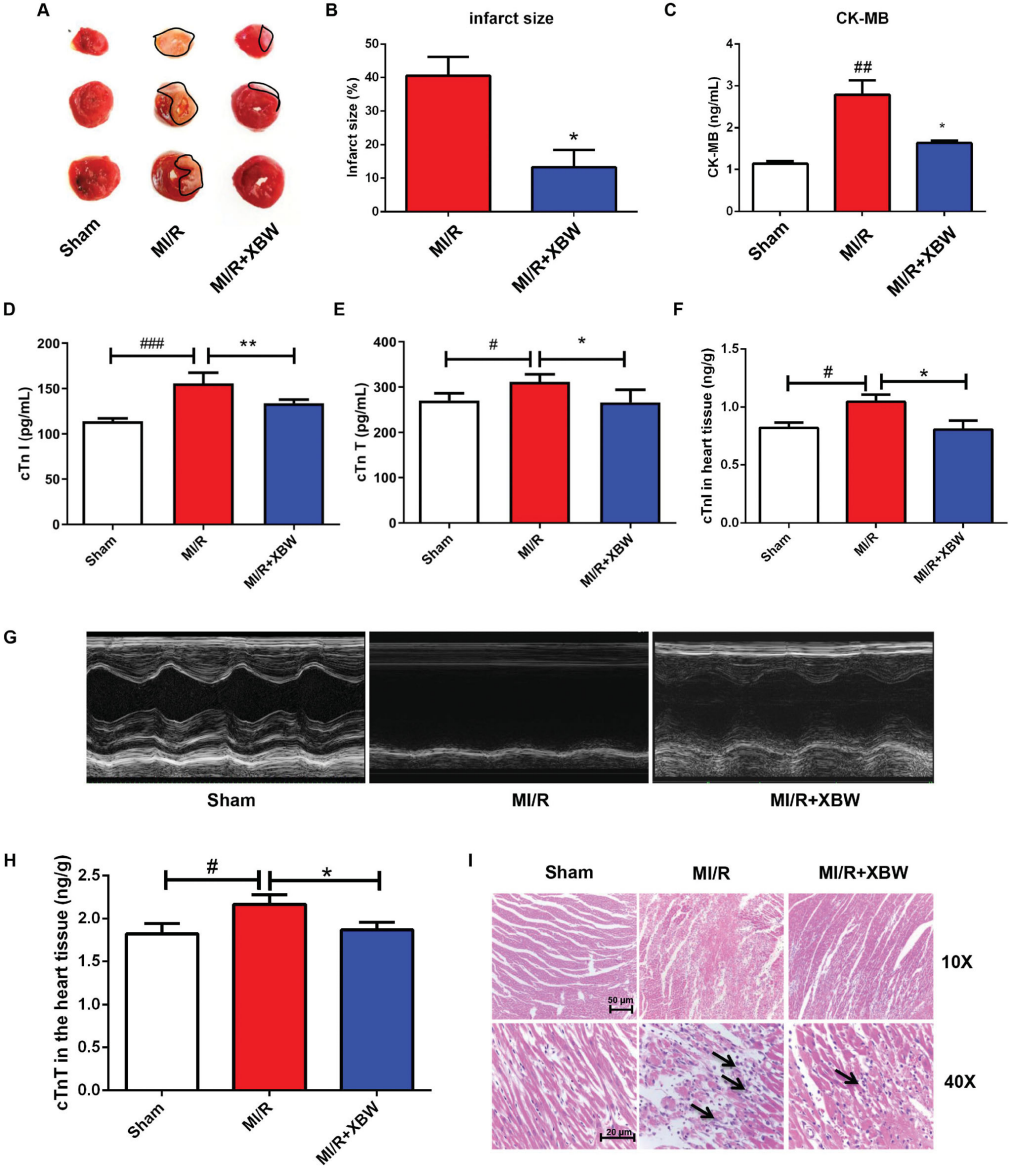
XBW administration attenuated MI/RI in LAD-induced rat model. (A) Representative images of TTC staining for the myocardial infarct size. (B) XBW decreased myocardial infarct size. *n* = 6, *p< 0.05, MI/R group vs. MI/R + XBW group. (C–E) XBW reduced plasma CK-MB, cTnI and cTnT levels in rats. *n* = 4–6, CK-MB, cTnI and cTnT levels in plasma were measured by ELISA. Results were expressed as the mean ± SEM. ^#^*p* < 0.05, ^##^*p* < 0.01, ^###^*p* < 0.001, MI/R group vs. Sham group; *p< 0.05, **p < 0.01, MI/R group vs. MI/R + XBW group. (F and H) XBW reduced cTnI and cTnT levels in heart tissue. (G) Representative images of M-mode echocardiography from each group. (I) Effects of XBW on cell morphology and HE staining.

**Figure 4.**
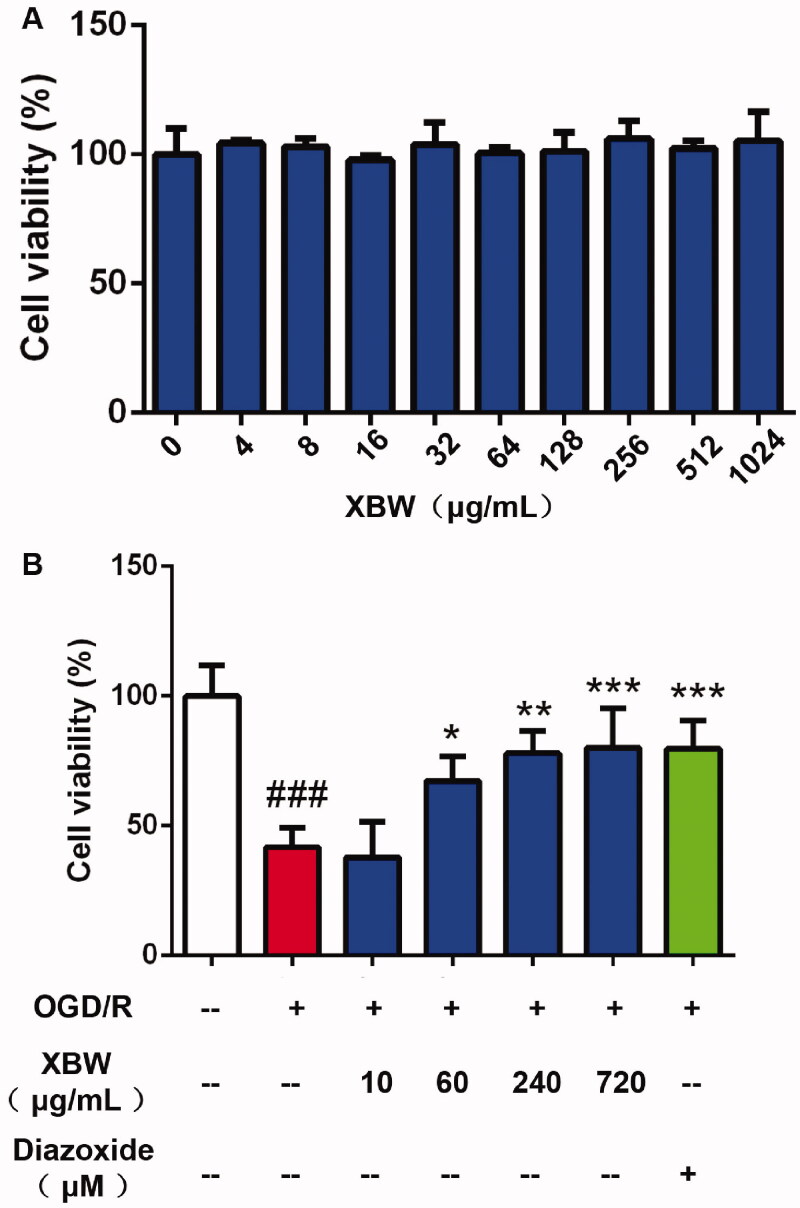
XBW protected H9c2 cells against OGD/R injury. (A) The cytotoxicity of XBW on H9c2 cells. H9c2 cells were treated with indicated doses of XBW (0, 4, 8, 16, 32, 64, 128, 256, 512 and 1024 μg/mL) for 48 h and determined by the MTT assay. (B) The effects of XBW on OGD/R-induced H9c2 cells. H9c2 cells were treated with indicated doses of XBW (0, 10, 60, 240 and 720 μg/mL) and diazoxide (100 μM), and followed by 6 h ODG condition and 18 h reperfusion. Cell viability was measured by the MTT assay. Results were expressed as mean ± SD, *n* = 4. ^###^*p* < 0.001, OGD/R group vs. Ctrl group; *p < 0.05, **p < 0.01, ***p < 0.001, XBW group vs. OGD/R group.

LAD ligation-induced MI/RI rat model was used to investigate the cardioprotective effect of XBW. As shown in Figure 3(A,B), XBW administration significantly reduced infarct size from 40.64% to 8.79%. Creatine kinase MB (CK-MB) is one of the biomarkers in serum for MI/RI, and XBW administration decreased the level of CK-MB induced by MI/RI from 2.83 to 1.63 U/L in plasma (Figure 3(C)). Meanwhile, the levels of cTnI and cTnT in plasma were also decreased from 155.8 ± 5.880 to 133.9 ± 2.047 pg/mL (*p* < 0.01), from 311.5 ± 7.663 to 266.6 ± 15.81 pg/mL (*p* < 0.05), respectively (Figure 3(D,E)). Figure 3(F, G) also shows that XBW could decrease the level of cTnI and cTnT in the heart tissue from 1.047 ± 0.061 to 0.808 ± 0.078 ng/g (*p* < 0.05) and from 2.166 ± 0.111 to 1.868 ± 0.092 ng/g (*p* < 0.05). Echocardiography in Figure 3(H) exhibited that XBW improved cardiac function. H&E staining showed that XBW administration attenuated inflammatory cell infiltration, and disordered myocardial fibre induced by MI/RI (Figure 3(I)). In *in vitro* study, XBW protected H9c2 cell against OGD/R injury from 40.08% to 58.8%, 77.9%, 80.1% at 60, 240 and 720 μg/mL, respectively (Figure 4(A,B)). These results suggested that XBW ameliorated MI/RI.

### Target identification and network analysis

**Figure 5.**
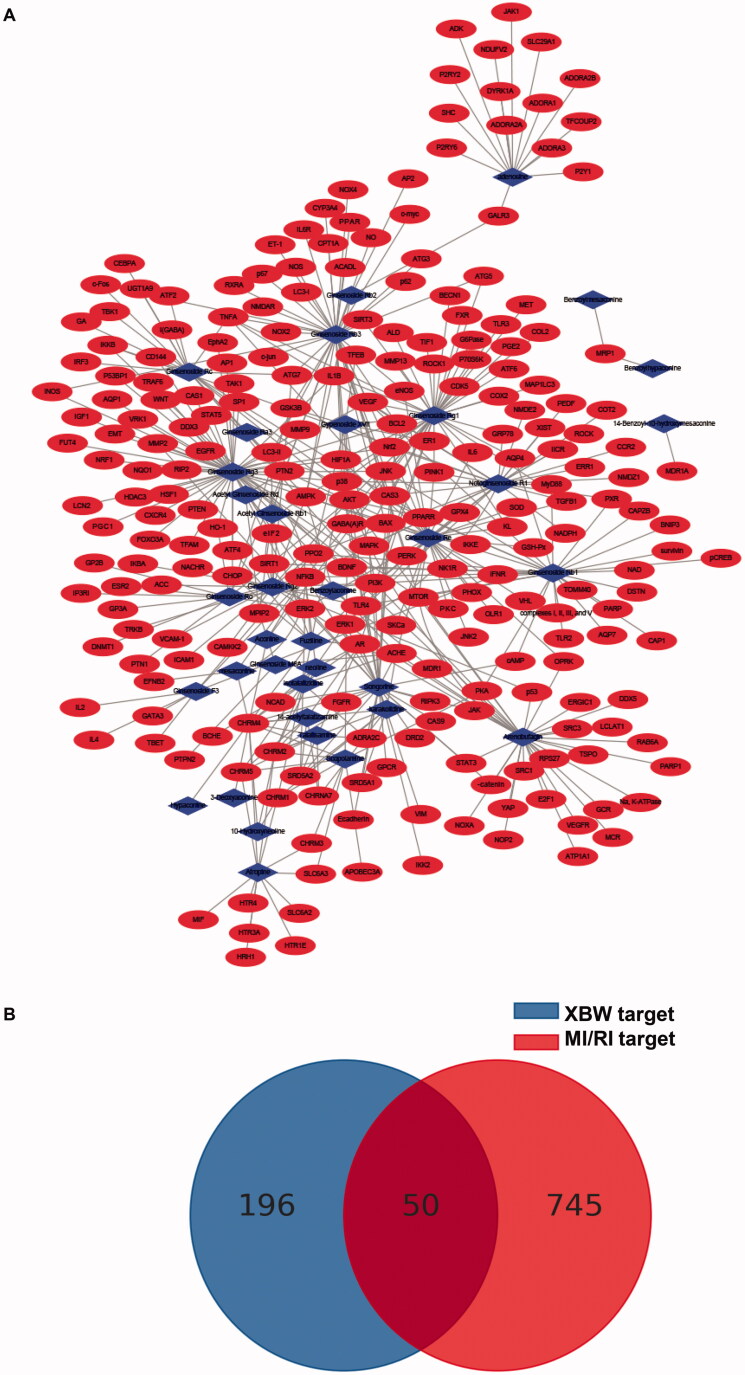
Network of compound and disease targets. (A) Compound-target network. Network of 37 compounds from XBW and 246 putative targets. (B) The common targets of compound targets and MI/RI targets.

Using TargetNet database and literature reported targets, we obtained 246 targets of 37 compounds in XBW (Figure 5(A)). The network contained 283 nodes and 462 edges, and its average number of neighbours was 3.216. After crossing with MI/RI targets, 50 targets were identified and a compound-target network is constructed in Figure 5(B,C).

**Figure 6.**
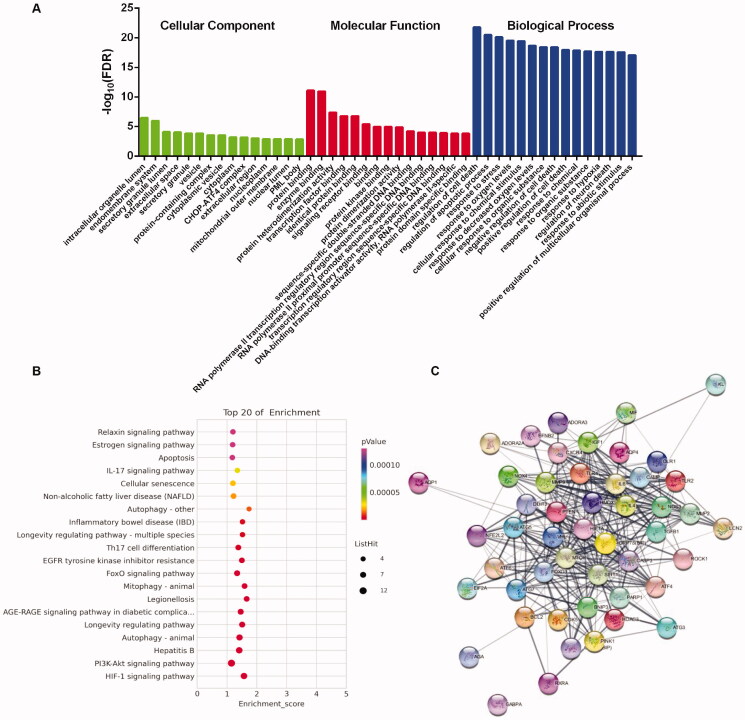
The GO and KEGG analysis. (A) The GO CC-MF-BP analysis diagram. (B) The KEGG analysis diagram. (C) PPI network graph of 50 hub Nodes based on their interactions.

After GO enrichment analysis of the targets by the String database, the top 15 enrichment results listed in BPs, MFs and CCs are shown in Figure 6(A), which indicated that XBW may regulate the apoptosis and stress response of cardiomyocytes via protein binding, enzyme binding, transcription factor binding, protein kinase binding, extracellular space, CHOP-ATF4 complex, etc. to attenuate MI/RI. To clarify the underlying pathways of XBW on MI/RI, KEGG pathway analysis is performed in Figure 6(B), which exhibited the top 20 related signalling pathways excluding the specific cancer related pathways, HIF-1 signalling pathway, PI3K-Akt signalling pathway, autophagy, FoxO signalling pathway, apoptosis, etc. Based on the protein–protein interactions (PPI) analysis (Figure 6(C)), CASP3, MTOR (rapamycin), SIRT1, HIF1A, ATF4, GRP78 (BIP, glucose regulated protein 78) and ATG7 were identified with high-degree targets, which played important roles in apoptosis, autophagy and endoplasmic reticulum (ER) stress.

### Experimental validations of the molecular mechanisms of XBW against MIRI

**Figure 7.**
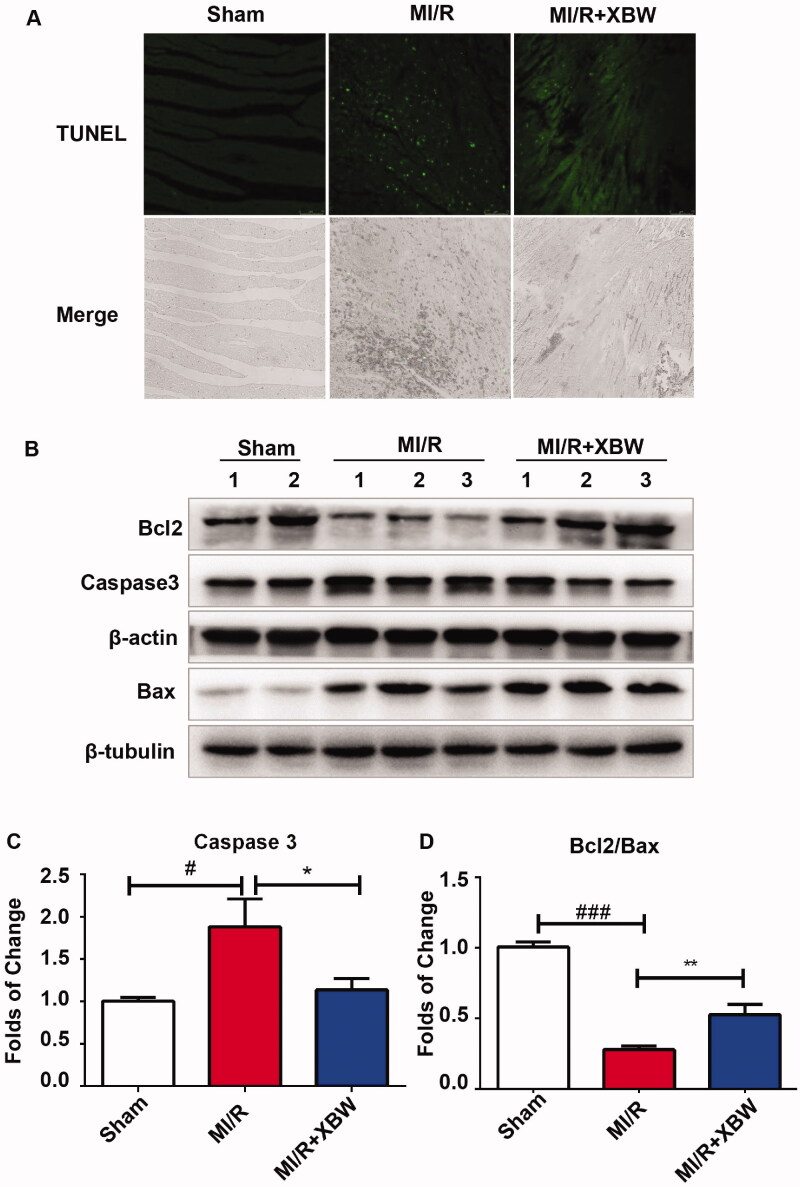
XBW suppressed myocardial apoptosis in rat with MI/RI. (A) TUNEL staining for apoptosis cells. (B–D) The effect of XBW on apoptosis-related protein expressions. All results are expressed as the mean ± SEM. *n* = 5–6, ^#^*p* < 0.05, ^###^*p* < 0.001, Sham vs. MI/R group; *p < 0.05, **p < 0.01, MI/R + XBW vs. MI/R group.

Cardiomyocyte apoptosis is a key factor in the pathological process of MI/RI. As shown in Figure 7(A), after 24 h reperfusion, TUNEL staining showed the percentage of apoptosis-positive cells in MI/RI group increased, while decreased in XBW treatment group. In addition, the apoptosis related proteins were detected. XBW increased the ratio of Bcl2/Bax expression from 0.2799 ± 0.0258 to 0.5273 ± 0.0735-fold (*p* < 0.01) and decreased caspase 3 (CASP3) expression from 1.883 ± 0.3307 to 1.138 ± 0.1334-fold (*p* < 0.05) (Figure 7(B–D)).

**Figure 8.**
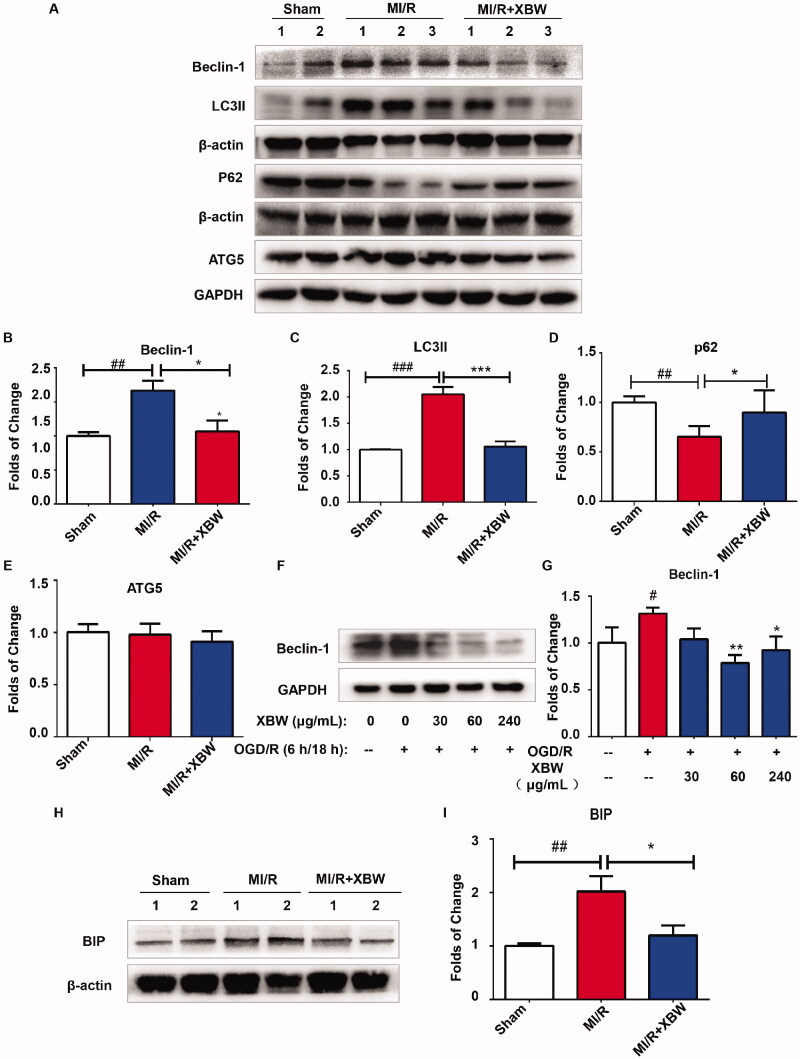
XBW prevented excessive autophagy and ER stress under MI/RI *in vivo* and *in vitro* models. (A–E) The expression of Beclin-1, LC3II, p62 and ATG5 in heart tissue. After MI/RI, the heart tissues in each group were collected and subjected to Western blotting analysis. *n* = 5, *^##^p* < 0.01, ^###^*p* < 0.001, Sham vs. MI/R group; *p < 0.05, **p < 0.001, ***p < 0.001, MI/R + XBW vs. MI/R group. (F, G) The expression of Beclin-1 in H9c2 cells. H9c2 cells were treated with indicated doses of XBW (0, 30, 60 and 240 μg/mL), and followed by 6 h ODG condition and 18 h reperfusion. Protein of H9c2 cells was isolated and subjected to Western blotting. *n* = 3, ^#^*p* < 0.05, Ctrl vs. OGD/R; *p < 0.05, **p < 0.001, OGD/R + XBW vs. OGD/R. (H, I) The expression of BIP in heart tissue. After MI/RI, the heart tissues in each group were collected and subjected to Western blotting analysis. *n* = 5, ^##^*p* < 0.01, Sham vs. MI/R group; *p < 0.05, MI/R + XBW vs. MI/R group.

During MI/RI, autophagy was also activated following MI/RI in cardiomyocytes to involve in the apoptosis of myocardial cells (Tannous et al. 2008; Wu SY et al. 2018). Beclin-1, LC3II, p62 and ATG5 expressions were detected (Figure 8(A–E)). The expressions of Beclin-1 and LC3II in cardiac tissue were markedly increased in MI/RI group, while significantly decreased in XBW administration group from 1.668 ± 0.143 to 1.067 ± 0.161-fold (*p* < 0.05) and from 2.053 ± 0.137 to 1.060 ± 0.100-fold (*p* < 0.001). Meanwhile, the expression of p62 was reduced in the MI/RI group, while increased in XBW group from 0.653 ± 0.044 to 0.899 ± 0.100-fold (*p* < 0.05). However, ATG5 was no obvious changes between three groups. *In vitro* study, XBW could also decrease Beclin-1 expression induced by OGD/R in H9c2 cells (Figure 8(H,G)). Cardiomyocytes open the unfolded protein response triggered by ER stress as a defensive mechanism at early stage of MI/RI; however, excessive ER stress induced cell apoptosis or even death. As shown in Figure 8(H,I), the expression of BIP, a marker of ER stress, was significantly increased in MI/RI group compared with that in the Sham group; however, XBW administration reduced the elevation of BIP expression. Taken together, these data demonstrated that the cardioprotective effect of XBW against MI/RI was associated with the attenuation of ER stress and autophagy.

## Discussion

Myocardial ischaemia–reperfusion injury is a difficult clinical problem in myocardial infarction therapy; however, current medications for treating MIRI are not ideal (Thind et al. 2015). TCM exhibits unique advantages in the treatment of cardiovascular diseases, based on multiple components and multiple targets (Hao M et al. 2017; Hao P et al. 2017). XBW is a patented traditional Chinese herbal formula, which has been listed in China for more than 30 years. It is used for treating ischaemic heart disease and chronic heart failure (He et al. 2020). However, there is a lack of evidence for the material basis and underlying mechanism of XBW against MI/RI. In the current study, we integrated chemical profile, network pharmacology, pharmacology and molecular cell biology to investigate the cardioprotective effect and mechanism of XBW against MI/IR.

XBW has been used to treat coronary heart disease and chronic heart failure (Li et al. 2018). In our study, an *in vivo* MI/RI rat model was used by performing LAD. The results showed that XBW administration remarkably decreased MI/RI-induced myocardial infarct size and improved cardiac left ventricular (LV) function. Moreover, the *in vitro* results revealed that XBW could also reduce OGD/R-induced cell injury. XBW is composed of nine Chinese medicines. We used UPLC-Q-TOF-MS/MS method to identify 37 chemical constitutes in Table 1, which provided the information of material basis. Importantly, most of these components were from *Panax ginseng* and Fuzi, which are monarch drugs in XBW. For example, *Panax ginseng* has the effects of invigorating Qi, promoting tissue regeneration and enhancing human body resistance (Kim 2018). Fuzi possesses the effects of causing restoration from collapse, reinforcing fire and Yang, and is used to treat acute myocardial infarction and chronic heart failure (Zhou et al. 2015). Consistently, reports have also showed that *Panax ginseng*, ginsenoside Rg3 and notoginsenoside R1 can alleviate MI/RI by suppressing oxidative stress, apoptosis, inflammation and regulating myocardial energy metabolism (Luo et al. 2013; Zhang et al. 2016; Tong et al. 2019). Fuzi and its alkaloids can improve inotropic effect, LV diastolic function (Liu et al. 2012), and energy metabolism (Yu et al. 2012), scavenge hydroxyl radicals and suppress lipid peroxidation to show the cardioprotective effects (Wu et al. 2019). Moreover, network pharmacological analysis of XBW identified that ginsenoside Rg3, Rg1, Rb3, arenobufagin and notoginsenoside R1 had high degrees, which may be the active compounds of XBW. Importantly, it is reported that notoginsenoside R1 attenuated MIRI by inhibiting oxidative stress- and ER stress-related signalling pathways (Yu LM et al. 2016; Yu YL et al. 2016). Multiple evidence has shown that ginsenoside Rg1 protected heart against MIRI partially by activating PI3K/Akt/mTOR and inhibiting autophagy (Zhang et al. 2012; Qin et al. 2018). Ginsenoside Rb3 and Rg3 also improved cardiac functions and protected MIRI via suppressing apoptosis and inflammation (Liu et al. 2014; Zhang et al. 2016). The results of the studies were consistent to our network pharmacological analysis. Although some active components have been reported in MI/RI, it still has several compounds without pharmacological verification, thus, their pharmacological effects need to be verified in the future work.

After screening with MI/RI-related proteins, 50 putative targets of XBW were collected. Among them, CASP3 activation is a biochemical hallmark of apoptosis (Choudhary et al. 2015); B cell lymphoma-2 (BCL2) plays an important role in the negative regulation of apoptosis during MI/IR (Huang et al. 1997; Wang G et al. 2016); eukaryotic initiation factor 2 alpha (eIF2α) and activating transcription factor-4 (ATF4) can mediate myocardial ER stress (Yu LM et al. 2016; Yu YL et al. 2016). mTOR and Beclin-1 are two key autophagy-related proteins in MI/R injury (Shi et al. 2019). The results illustrated that XBW might regulate above proteins to show cardioprotective effect. GO enrichment analysis showed that XBW can treat MI/RI by regulation of cell death, apoptosis process and response to stress. KEGG enrichment analysis demonstrated that apoptosis, autophagy, HIF-1 signalling pathway, PI3K/Akt signalling pathway and FoxO signalling pathway were involved in XBW for treating MI/RI. To further validate the prediction and analysis, we investigated the key potential mechanism of XBW against MI/RI *in vitro* and *in vivo*.

Reducing cardiomyocyte death and infarct size is necessary to MI/RI. Myocardial apoptosis is a key factor for the most of cell death during cardiac pathological processes of MI/RI, while blocking the apoptosis-related signalling pathways helps prevent myocardial injury (Jennings 2013; Zhu et al. 2015). Anti-apoptotic protein Bcl-2 and pro-apoptotic protein Bax are involved in the stage of apoptosis. In the present study, XBW suppressed myocardial apoptosis with the decreased TUNEL positive cells and the increased ratio of Bcl2/Bax expression. Therefore, XBW has the potential effects for attenuating myocardial apoptosis for the patients with MI/RI.

Emerging evidence has indicated that ER stress is involved in the development and pathogenesis of MI/RI (Huang et al. 1997; Wang J et al. 2016). During MI/RI, the balance of the homeostasis for the ER is broken, subsequently unfolded or misfolded proteins are accumulating in myocardial cells, and eventually triggering ER stress (Wu et al. 2016). At the early stage, a certain degree of ER stress helps self-repair injured cells; however, if ER stress is excessive, it will provoke the apoptotic signalling pathway activation (Li et al. 2019). GRP78 (BIP) is a calcium ion-binding molecular chaperone in the ER. When undergoing ER stress, GRP78 and ER cross to activate the downstream CHOP-associated apoptotic signalling pathways (Wu LX et al. 2018). In our study, XBW treatment decreased the expression of BIP induced by MI/RI *in vivo* or OGD/R injury *in vitro*. Autophagy has a dual function in MI/RI (Nishida et al. 2009). Several studies showed that reduction of autophagy clearance in myocardial cells during MI/RI threatens cell survival (Ma et al. 2012; Hao M et al. 2017; Hao P et al. 2017). Promoting autophagy moderately may protect cell and mitochondrial injury in MI/RI (Tannous et al. 2008; Wu SY et al. 2018). However, at the late stage of MI/RI, it induces excessive activation of autophagy, resulting in cytotoxic cell death (Zhu et al. 2007; Kroemer and Levine 2008; Huang et al. 2017). Thus, prevention of excessive autophagy activated during MI/RI may be benefit to reduce cardiomyocyte death and improve cardiac function. In the present study, XBW treatment inhibited MI/RI-induced beclin-1 and LC3II expression to inactivate excessive autophagy. These results fully demonstrated that XBW protected heart against MI/RI through the multicomponent, multitarget and multipathway. The pathogenesis of MI/RI is complex, and XBW has potential clinical application value for the prevention and treatment of multiple pathways.

However, there are still several limitations in our study to be solved in the future work. By using UPLC-Q-TOF-MS/MS, 37 major compounds from six medicinal materials were identified, but the volatile constituents of three others, including *Cinnamomum cassia* Presl, moschus and Borneolum syntheticum had not been detected, which needs QC–MS analysis in our later work to enrich material basis of XBW. In addition, the present study only evaluated the overall efficacy and mechanism of XBW, but the effects and underlying mechanism of the identified active compounds, such as ginsenoside Rg3, Rg1, Rb3, etc., have to be further verified.

## Conclusions

**Figure 9.**
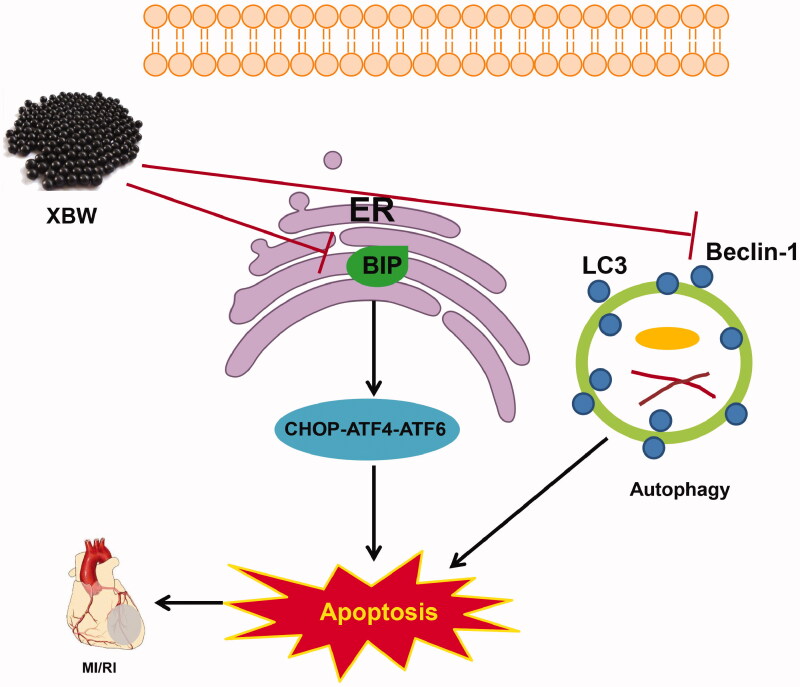
Overview of potential mechanism of XBW against myocardial ischaemia–reperfusion injury. XBW showed therapeutic effects against MI/RI mainly via attenuating apoptosis through suppressing excessive autophagy and ER stress.

In the present study, we revealed the therapeutic effect and underlying mechanism of XBW against MI/IR based on chemical profile, network pharmacology and experimental support. Thirty-seven chemical constituents in XBW were identified, 50 potential MI/RI targets and five significant pathways were achieved by network pharmacology analysis. Collectively, our results demonstrated that XBW ameliorated the apoptosis of cardiomyocytes in MI/RI by suppressing autophagy and ER stress (Figure 9).

## Author contributions

YC and ZL designed the experiments, conducted and revised the manuscript. YY, TC, JL and SC performed the experiments and wrote the manuscript. RC, LW, JH, QL and XQ collected and partially analysed the data. All authors reviewed and revised the manuscript.
